# Switching and optimizing control for coal flotation process based on a hybrid model

**DOI:** 10.1371/journal.pone.0186553

**Published:** 2017-10-17

**Authors:** Zhiyong Dong, Ranfeng Wang, Minqiang Fan, Xiang Fu

**Affiliations:** College of Mining Engineering, Taiyuan University of Technology, Taiyuan, Shanxi, China; Southwest University, CHINA

## Abstract

Flotation is an important part of coal preparation, and the flotation column is widely applied as efficient flotation equipment. This process is complex and affected by many factors, with the froth depth and reagent dosage being two of the most important and frequently manipulated variables. This paper proposes a new method of switching and optimizing control for the coal flotation process. A hybrid model is built and evaluated using industrial data. First, wavelet analysis and principal component analysis (PCA) are applied for signal pre-processing. Second, a control model for optimizing the set point of the froth depth is constructed based on fuzzy control, and a control model is designed to optimize the reagent dosages based on expert system. Finally, the least squares-support vector machine (LS-SVM) is used to identify the operating conditions of the flotation process and to select one of the two models (froth depth or reagent dosage) for subsequent operation according to the condition parameters. The hybrid model is developed and evaluated on an industrial coal flotation column and exhibits satisfactory performance.

## Introduction

In the process of coal preparation, flotation is used to separate the ash-forming mineral matter and the carbonaceous materials of fine coal below 0.5 mm in size [1,2]. This process is widely applied in countries such as China, USA, Australia, Canada and India [3]. Flotation columns are extensively studied and used as an efficient coal flotation equipment due to many advantages, including the simplicity of construction, lack of moving parts, and low energy consumption, among others. [4]. According to the different structures and separation principles, flotation columns mainly include the Leeds column, Microcel column, packed column, Flotaire column, hydrochem column, Jameson column and cyclonic micro-bubble column [5].

Over the past two decades, increasing attention has been focused on research on intelligent control of the flotation process. Supervisory control and multivariate projection methods have been successively applied to flotation columns [6]. Ou et al. described a multiple input/multiple output (MIMO) control system using a method that combined PID and fuzzy control for the flotation column in which the two output variables were the aeration rate and pulp level [7]. Mohanty used an artificial neural network to achieve predictive control of the flotation pulp level [8]. Maldonado et al. used a combined PI and multivariable predictive control strategy to control the flotation column [9]. Karelovic et al. proposed a framework for predictive control of a hybrid model in mineral processing [10], and Putz and Cipriano applied the hybrid model predictive control to flotation circuits, demonstrating performance superior to that of conventional schemes [11]. Xie et al. proposed an integrated control strategy for reagent addition, and the model included an estimator of the feed grade based on probabilistic support vector regression, a preset controller based on the operational pattern method, and a feedback controller based on the fuzzy control system [12]. These studies indicate that hybrid control methods are suitable and effective for optimization of the flotation process.

Cubillos and Lima showed that reagent dosage and pulp level are two primary variables manipulated in the flotation process [13]. These two variables are also the most frequently adjusted in the industrial coal flotation process and directly affect the quality of flotation products. Expert systems and fuzzy supervisors have proven to be practical and excellent tools for complex processes [14]. A hybrid system combining expert systems and fuzzy controllers has been used at the El Teniente Concentrator and the Salvador Concentrator [15], and a feedforward and feedback expert system (FFFBES) that combines a feedforward action with classical feedback expert control was successfully implemented in an experimental flotation circuit [16]. The research presented in this work uses fuzzy control for set-point optimization of the froth depth and the expert system for set-point optimization of the reagent addition.

Although both the froth depth and reagent dosage significantly influence the result of flotation, these variables are typically not adjusted simultaneously due to the coupling between variables and the hysteresis of the flotation process. For coal preparation plants that contain a distributed control system (DCS), operators typically determine which variable must be adjusted in real time and change the set point according to observation and experience. In the actual operation process, with the aim of ensuring product quality, we prefer to reduce the reagent dosage rather than adjust the froth depth, and we prefer to adjust the froth depth rather than increase the dosage. The coordinated adjustment of these two variables is the key to ensuring the quality of the flotation products and reducing the costs. To select an appropriate controller within a reasonable time frame, the hybrid model should be able to execute a reasonable and timely switch of the controllers using auxiliary variables. Thus, controllers for different manipulated variables are used according to different condition parameters. In this case, the determination of switching points is critical.

The support vector machine (SVM) proposed by Vapnik [17] has been widely developed in pattern recognition, image classification, regression prediction and other fields [18,19]. In the least squares-support vector machine (LS-SVM), the equality constraints are used to replace the inequality constraints of SVM [20]; thus, the computational complexity is reduced, and the calculation speed is greatly accelerated. Therefore, the LS-SVM modeling method can meet the actual requirements of the industrial process. In the work presented, the LS-SVM is used in identification and classification of the operating conditions of the floatation process, and a selection is made between control of the froth depth or reagent addition.

In the process of modeling, data pre-processing plays an important role in the accuracy of the model. In this research, the modeling data are obtained from sensors in the actual industrial process such that the measurement inevitably contains a certain amount of outliers and noise. The accuracy and reliability of the data can be guaranteed by filtering. Wavelet filtering, which is conducted in many fields and offers favorable results [21,22], is adopted in this study. In addition, principal component analysis (PCA) is widely applied to extract features and reduce dimensions [23,24]. Because many variables affect the flotation process, it is challenging to estimate which features are more sensitive to the model identification results. Therefore, in this study, PCA is used to fuse the original features and extract the more sensitive features for use as the input of the LS-SVM.

**Fig 1:**
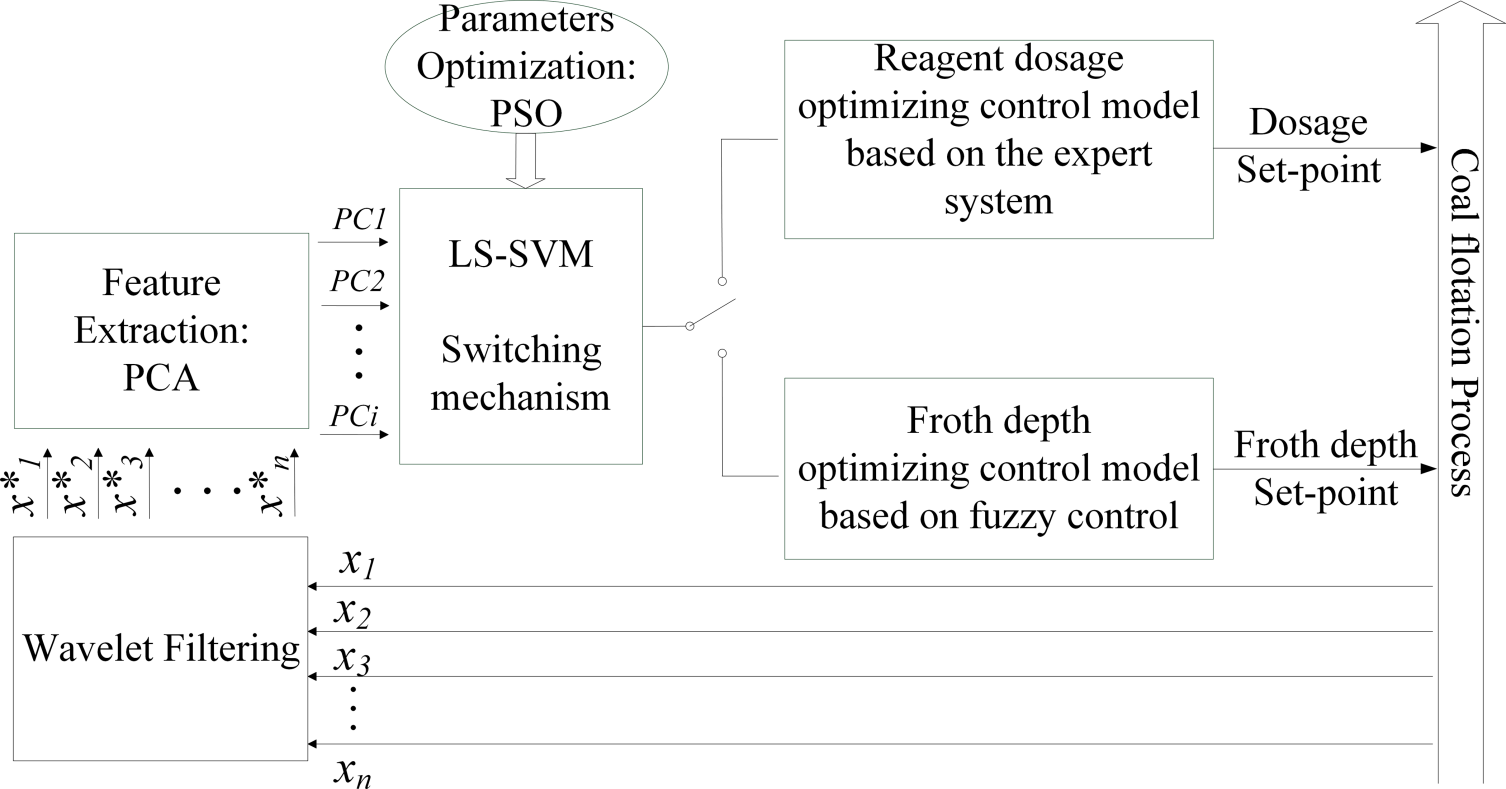
Structure of the proposed hybrid model.

The remainder of this paper is organized as follows. The coal flotation process is described in Section 2, and the modeling approaches are introduced in Section 3. In Section 4, we report the results of the experiments and evaluation of the proposed model. Section 5 presents the conclusions of this research. The structure of the proposed hybrid model is shown in Fig 1.

## Process description

**Fig 2:**
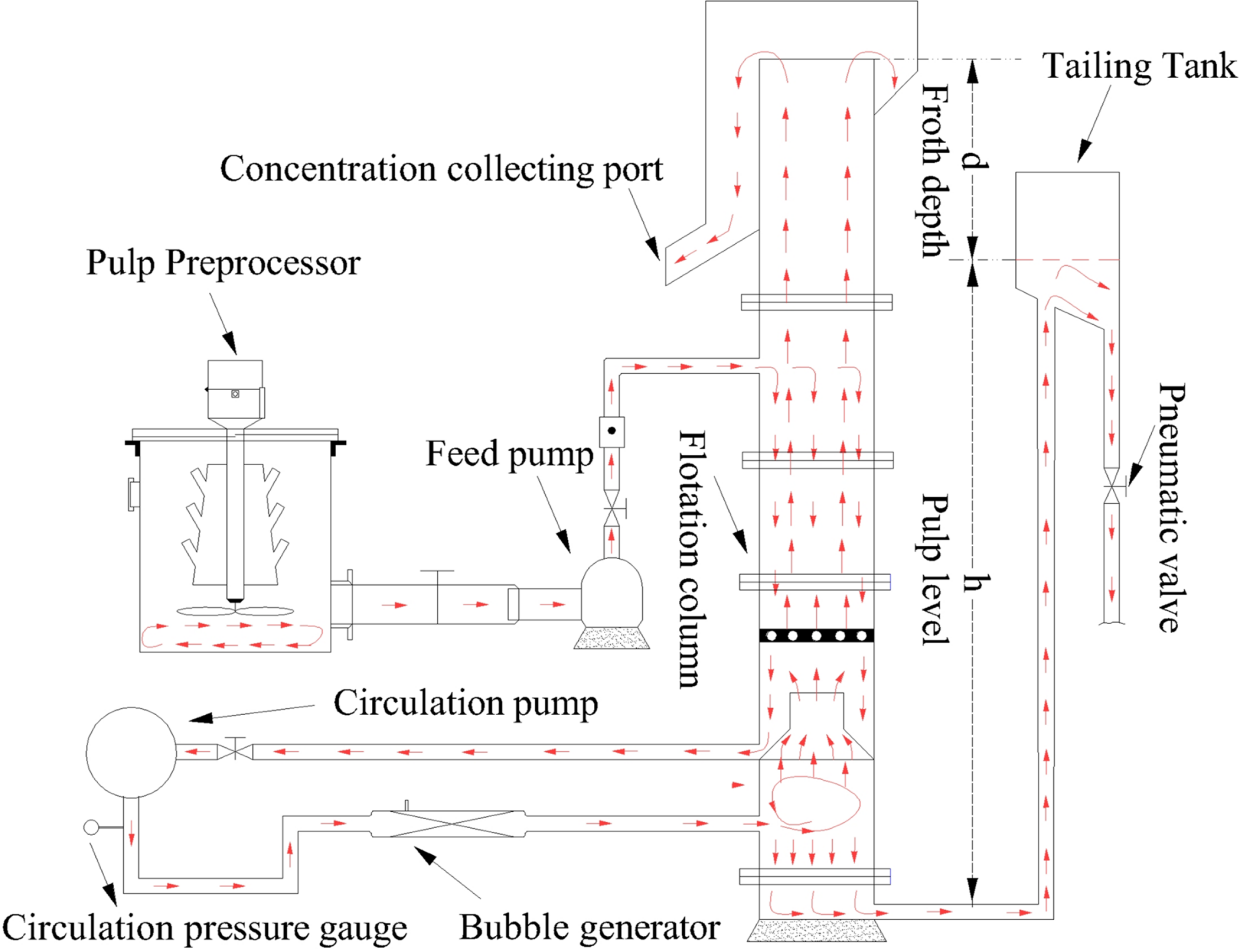
Schematic illustration of the FCMC flotation column [2].

For research on switching and optimizing control for the coal flotation process, we chose a coal preparation plant that uses a cyclonic micro-bubble flotation column (abbreviated as FCMC), which was developed and patented by Liu, J. T. [25] and is widely used in Chinese coal preparation plants. A schematic illustration of the flotation column is shown in Fig 2 [2]. The FCMC flotation column is divided into three working zones: the froth zone, collection zone and scavenging zone. The washing device and overflow groove are located on the top of the column. The inlet is located at approximately one third of the column height. The concentrate is discharged from the overflow groove, and the tailings are discharged from the underflow port. The circulation pump is connected to the air bubble generator and is situated outside the column body. When the circulating pump jets the slurry, the bubble generator inhales air and mixes it with the frother in the coal slurry. A large number of micro-bubbles are released in the pressure-reduction process. Micro-bubbles enter the column along the tangential direction and move rotationally under centrifugal force. The bubbles and mineralized gas-solid floccules move upward through the rotational flow center and enter the collection zone. The unmineralized tailings move downward and discharge through the underflow. The reverse movements of the feed and air bubbles promote the mineralization and formation of gas-solid floccules.

The process of FCMC flotation column has the following main effect factors: column height, particle size distribution, feed ash content, concentration, flow rate, gas holdup, wash water rate, reagent dosage, and froth depth (pulp level) [26,27]. Every factor has a certain effect on the quality of the coal flotation products (ash content and recovery). The gas holdup is determined by the frother dosage and the circulating pulp pressure [28]. The particle size compositions differ between coal flotation and ore floatation, and the fine particle content of coal flotation is less than that of ore floatation. Additionally, according to the manufacturer’s instructions, under the premise of the product quality is qualified, as little wash water as possible should be used and even without use of wash water. Therefore, the wash water rate is not considered in this paper. Reasonable reagent addition can promote the separation of useful minerals and gangue by improving the hydrophobicity of the minerals [29]. The froth depth also plays an important role. In a flotation column, the particles collide with the bubbles and adhere to them in the collection zone. The froth zone is composed of the bubbles that adhere to particles. By increasing the collection zone, the pulp level can increase the probability that particles stick to the bubbles, thus improving the recovery of the concentrate. In contrast, by increasing the froth zone, the froth depth can enhance the secondary enrichment of froth flotation, thus improving the grade of the concentrate. In this research, the froth depth and reagent addition are selected as the two manipulated variables. In the process of coal flotation, the main process variables include disturbance variables, manipulated variables, and controlled variables, as listed in Table 1.

**Table 1:** Process variables in the proposed hybrid model.

Variable type	Name
Disturbance variables	Feed concentration
	Feed flow rate
	Circulating pulp pressure
Manipulated variables	Collector dosage
	Frother dosage
	Froth depth
Controlled variables	Ash content of clean coal
	Recovery rate of clean coal

## Hybrid model establishment

In this work, we define two different control levels in automatic control of the flotation column, namely, stable control and optimizing control. The purpose of stable control is to ensure that the flotation column operates in a stable state under the fixed parameters and obtains stable and qualified products. Stable control is the basic requirement of flotation column automation. Based on stable control, optimization control can be applied to adjust the set points of the main manipulated variables according to different flotation processes. Under the premise of ensuring qualified products, the cost consumption is maintained at the lowest value.

### Optimizing control model for froth depth based on fuzzy control

**Fig 3:**
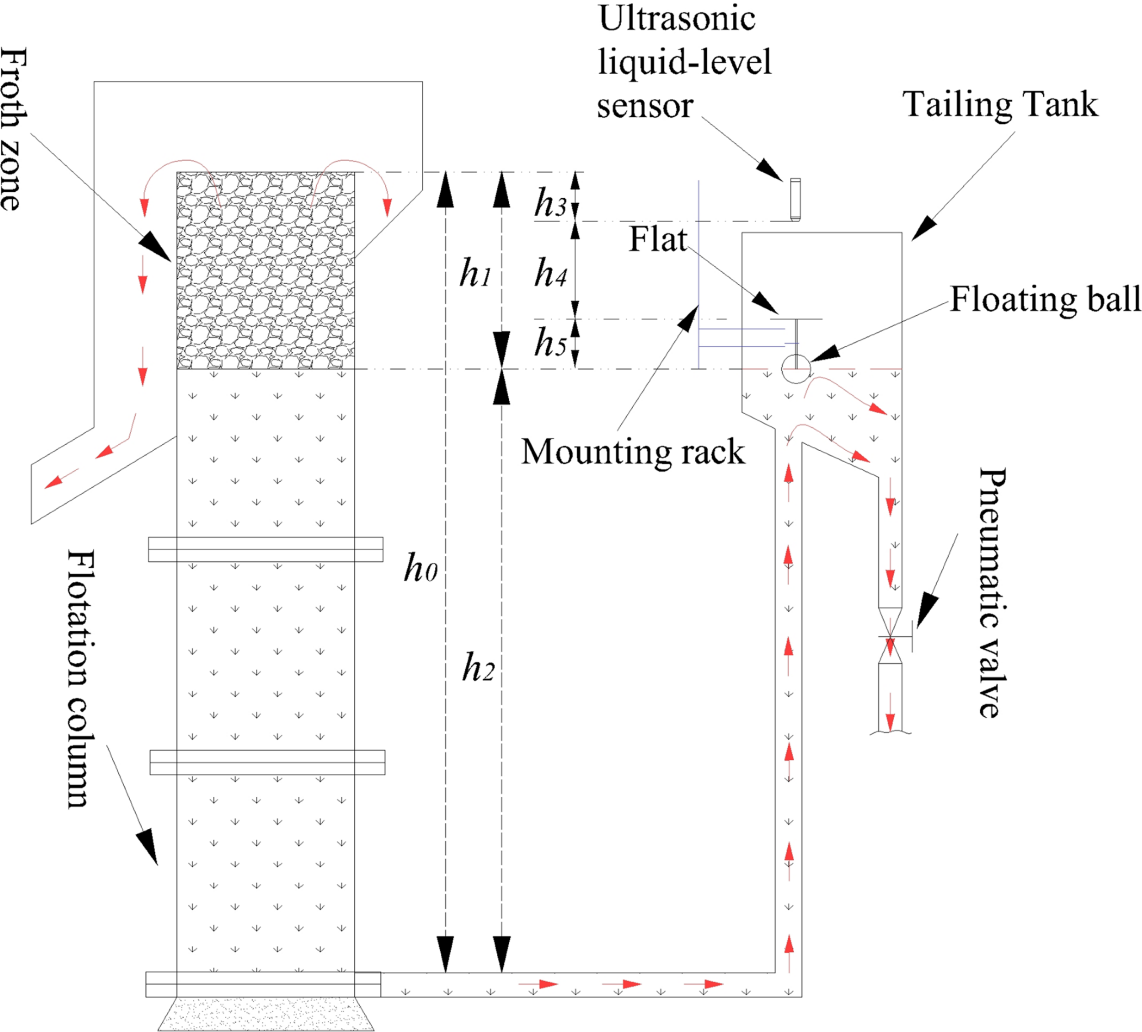
Schematic diagram of the froth depth measurement.

In the coal slime flotation process, the froth depth (pulp level) is one of the most frequently adjusted variables and has a significant influence on the performance of the flotation process. The froth depth measurement method is shown in Fig 3. An ultrasonic liquid-level sensor is installed on the top of the tailing tank, a floating ball is connected to the flat plate through a connecting rod, and these items are fixed on the tailing tank through the mounting rack. The froth depth and pulp level are calculated according to Eqs 1 and 2.

$$h_{1}=h_{3}+h_{4}+h_{5}$$ (1)

$$h_{2}=h_{0}-h_{1}$$ (2)

where *h*_*0*_ represents the flotation column height, *h*_*1*_ represents the froth depth, *h*_*2*_ represents the pulp level, *h*_*3*_ represents the vertical distance between the probe of the ultrasonic liquid-level sensor and the overflow weir of the flotation column, *h*_*4*_ is the sensor measurement value, and *h*_*5*_ is the vertical distance between the liquid surface and the flat.

In the process of stability control of froth depth, the measured value of the froth depth is compared with the set point, and after PID calculation, the valve opening is output to control the tailing discharge rate. Based on stability control [30], the froth depth set-point optimizing control model is designed based on fuzzy control. The formula for the dynamic balance of the liquid level in the container can be expressed as in Eq 3.

$$ROC=Q_{in}-Q_{out}$$ (3)

where Q_in_ represents the liquid inflow per unit time, Q_out_ represents the liquid outflow per unit time, and ROC represents the rate of change of liquid storage in a container. In this coal flotation process, the feed flow has a significant influence on the froth depth. The deviation of the feed flow and the rate of change of deviation are used as the input of the fuzzy controller, and the froth depth is the output. "E" represents the deviation of feed flow, "EC" represents the rate of change of deviation, and "D" represents the froth depth. Fuzzy sets of E, EC and D are described as {NB, NM, NS, ZO, PS, PM, PB}, where "NB" means negative big, "NM" means negative medium, "NS" means negative small, "ZO" means zero, "PS" means positive small, "PM" means positive medium, and "PB" means positive big.

The triangle function is selected as the membership function. The Barycenter method is used in defuzzification. The fuzzy rules are designed according to the following ideas: When the E is PB and the EC is PB, this state indicates that the feed flow increases significantly. At this time, the froth depth set point should be increased, and the pulp level must be decreased, thus reducing the overflow velocity of the froth, enhancing the secondary enrichment, and ensuring the concentrate grade. Furthermore, improvement in the tailing discharge rate can guarantee the treatment capacity of the flotation column. Otherwise, when the E is NB and the EC is NB, this state indicates that the feed flow decreases significantly, and at this point, the froth depth set point should be decreased and the pulp level increased to supply additional time for particles to adhere to the bubbles, thus improving the recovery of the concentrate. Ultimately, the concentrate grade is improved. Accordingly, the fuzzy rules are designed as shown in Table 2.

**Table 2:** Fuzzy rules of froth depth control.

D		EC						
		NB	NM	NS	ZO	PS	PM	PB
**E**	NB	NB	NB	NM	NM	NM	ZO	ZO
	NM	NB	NM	NM	NM	NS	ZO	ZO
	NS	NM	NM	NS	NS	ZO	PS	PS
	ZO	NM	NM	NS	ZO	PS	PM	PM
	PS	NS	NS	ZO	PS	PM	PM	PM
	PM	ZO	ZO	PS	PS	PM	PB	PB
	PB	ZO	ZO	PS	PM	PM	PB	PB

The fuzzy control rule set can be described using fuzzy relation matrix A:

$$A=⋃E_{i}\times {EC}_{j}\times D_{k}$$ (4)

At point *n*,

$$D=(E_{n}{\times EC}_{n})○A$$ (5)

### Optimizing control model of reagent dosage based on expert system

The main coal flotation reagents are the collector and frother. In general, experienced operators are often able to adjust the reagent dosage in a timely manner according to their own experience. Thus, in this research, the expert system is based on operator experience and expert advice. Production rules of the type "if A and B, then C" are used to establish the expert rule base. The expert rules are shown below.

Rule 1: IF Q < Q_1_ AND C < C_1_, THEN Q_c_ = Q_c 1_ AND Q_f_ = Q_f 1_
……
Rule *j*: IF Q < Q_1_ AND C_*j*-1_ < C < C_j_, THEN Q_c_ = Q_c *j*_ AND Q_f_ = Q_f *j*_
……
Rule *n* + 1: IF Q < Q_1_ AND C_*n*_ < C, THEN Q_c_ = Q_c *n* + 1_ AND Q_f_ = Q_f *n* + 1_
……
Rule _i*(*n* + 1)_: IF Q_*i*-1_ < Q < Q_*i*_ AND C_n_ < C, THEN Q_c_ = Q_c i*(*n* + 1)_ AND Q_f_ = Q _i*(*n* + 1)_
……
Rule (*i*-1)*(*n* + 1)+*j*: IF Q_*i*-1_ < Q < Q_i_ AND C_*j*-1_ < C < C_*j*_,THEN Q_c_ = Q_c (*i*-1)*(*n* + 1)+*j*_ AND Q_f_ = Q_(*i*-1)*(*n* + 1)+*j*_
Rule (*m* + 1)*(*n* + 1): IF Q > Q_*m*_ AND C > C_*n*_, THEN Q_c_ = Q_c (*m +* 1)*(n + 1)_ AND Q_f_ = Q_f (*m +* 1)*(*n* + 1)_

where "*m*", ''*n*", "Q_*i*_", "Q_*j*_", "Q_c_", and "Q_f_" are determined by experienced operators and experts, and *i =* 1,…,*m*; *j =* 1,…,*n*. When the feed flow rate and concentration change, the collector and frother dosages are automatically adjusted according to the expert rules.

### Switching mechanism

The operating parameters of the complex production process are often coupled, and simultaneous adjustment of the manipulated variables tends to reduce the stability and cause oscillation of the system. Different factors are crucial at different times; thus, reasonable adjustment of a manipulated variable at the correct time is highly important to maintaining the proper function of the flotation process and ensuring the quality of the product. In a practical production process, operators tend to adjust the individual manipulated variable (reagent dosage or froth depth), and after observing the adjustment effect, they adjust another variable if necessary. However, operators rarely adjust two variables simultaneously. The coordinated adjustment of the two variables is the key to ensuring the quality of the flotation products and reducing the costs.

In this work, we consider a group of data that, as a whole, describes an operating condition, namely a pattern. The process parameters that describe the current state of the process are generally divided into condition parameters and decision parameters. The parameter of the current production process that can be measured *x* = {*x*_1_,*x*_2_,*…*,*x*_*i*_} is defined as the condition parameter, and the parameter of the corresponding operating condition category *y* = {+1, −1} is defined as the decision parameter.

In this paper, the hybrid model can select different controllers according to different operating conditions (condition parameters), which are described above. The controller switching mechanism is converted into a supervised classification problem, and the LS-SVM is used to solve this problem.

The training set is *S*:

$$S:\;\{(x_{i,}y_{i}),x_{i}\in R^{n},y_{i}\in \{-1,+1\},\;,\;i=1,2,\ldots N\}$$ (6)

where {*x*_*i*_} indicates the input vector and *y*∈{−1,+1} represents the corresponding output vector.

The discriminant function is described as follows:

$${g(x)=\omega }^{T}∙x+b$$ (7)

The optimal classification hyperplane must meet the condition that **|**g(*x*)**|**≥1. Thus,

$$\left\{ \begin{array}{c} \omega ∙x_{i}+b\geq +1\;,\;if\;y_{i}=+1\\ \omega ∙x_{i}+b\leq -1\;,\;if\;y_{i}=-1 \end{array}\right.\;i=1,2,\ldots N$$ (8)

The classification decision function can be expressed as follows:

$$f(x_{i})=sgn(\omega^{T}∙\emptyset (x_{i})+b)$$ (9)

where *Φ*(·) is a nonlinear function that maps the input vector to the high-dimensional feature space, *x*_*i*_ is the support vector, *ω* is the weight vector, and b is a constant. Thus, the LS-SVM classification in the feature space can be converted into an optimization problem:

$$\left\{ \begin{array}{c} \mathrm{Min}\;\emptyset (\omega ,e)=\frac{1}{2}\omega^{T}\omega +\frac{\gamma }{2}\sum_{i=1}^{N}e_{i}^{2}\\ \begin{array}{c} y_{i}(\omega^{T}∙\emptyset (x_{i})+b)=1-e_{i};\;i=1,2,\ldots ,\;N \end{array} \end{array}\right.$$ (10)

where *Φ*(*ω*,*e*) is the objective function, *γ* is the penalty factor, and *e*_*i*_ is the slack variable.

To solve the above problems, the Lagrange multiplier *α*_*i*_ is introduced, and the constrained problem is transformed into an unconstrained problem:

$$L(\omega ,b,e;\alpha )=\emptyset (\omega ,e)-\sum_{i=1}^{N}\alpha_{i}(y_{i}(\omega^{T}∙\emptyset (x_{i})+b)+e_{i}-1)$$ (11)

According to the KTT condition of optimal system theory, the following equations apply:

$$w=\sum_{i=1}^{N}\alpha_{i}y_{i}\varphi (x_{i});\;\sum_{i=1}^{N}\alpha_{i}y_{i}=0;\;\alpha_{i}=\gamma e_{i}$$ (12)

Thus, the linear equations are obtained as follows:

$$\left[\begin{array}{cc} 0 & Y^{T}\\ Y & \Omega +\gamma^{-1}I \end{array}\right]\left[\begin{array}{c} b\\ \alpha \end{array}\right]=\left[\begin{array}{c} 0\\ \bar{1} \end{array}\right]$$ (13)

where *Y* = [*y*_*1*_,···,*y*_*N*_], ī = [1,···,1], *α*_*i*_ = [*α*_1_, *α*_2_,···, *α*_*N*_], and *Ω*_*il*_ = *y*_*i*_*y*_*l*_*Φ*(*x*_*i*_)^*T*^*Φ*(*x*_*l*_), (*i*,*l*, *=* 1,···,*N*). We define *K*(*x*,*x*_*i*_) = *Φ*(*x*)^*T*^*Φ*(*x*_*i*_), *i* = 1,···,*N*. *K*(*x*,*x*_*i*_) to represent the kernel function, and the classification decision function is obtained as follows:

$$f(x)=sgn(\sum_{i=1}^{N}\alpha_{i}y_{i}K(x,x_{i})+b)$$ (14)

The selection of the kernel function is critical in the process of LS-SVM modeling. The RBF has been frequently applied in classification due to its favorable learning ability, excellent nonlinear mapping performance and simple form [31,32]. Therefore, the RBF is selected as the kernel function of the LS-SVM. The RBF is described as follows:

$$K(x,x_{i})=exp\;\left[-\frac{{\left(x-x_{i}\right)}^{2}}{{2\sigma }^{2}}\right]$$ (15)

However, the prediction accuracy of the LS-SVM model is heavily dependent on the selection of its internal parameters. In this research, the particle swarm optimization (PSO) algorithm [33,34], a random search algorithm based on group collaboration, is used to optimize the parameters “γ” and “σ^2^”.

**Fig 4:**
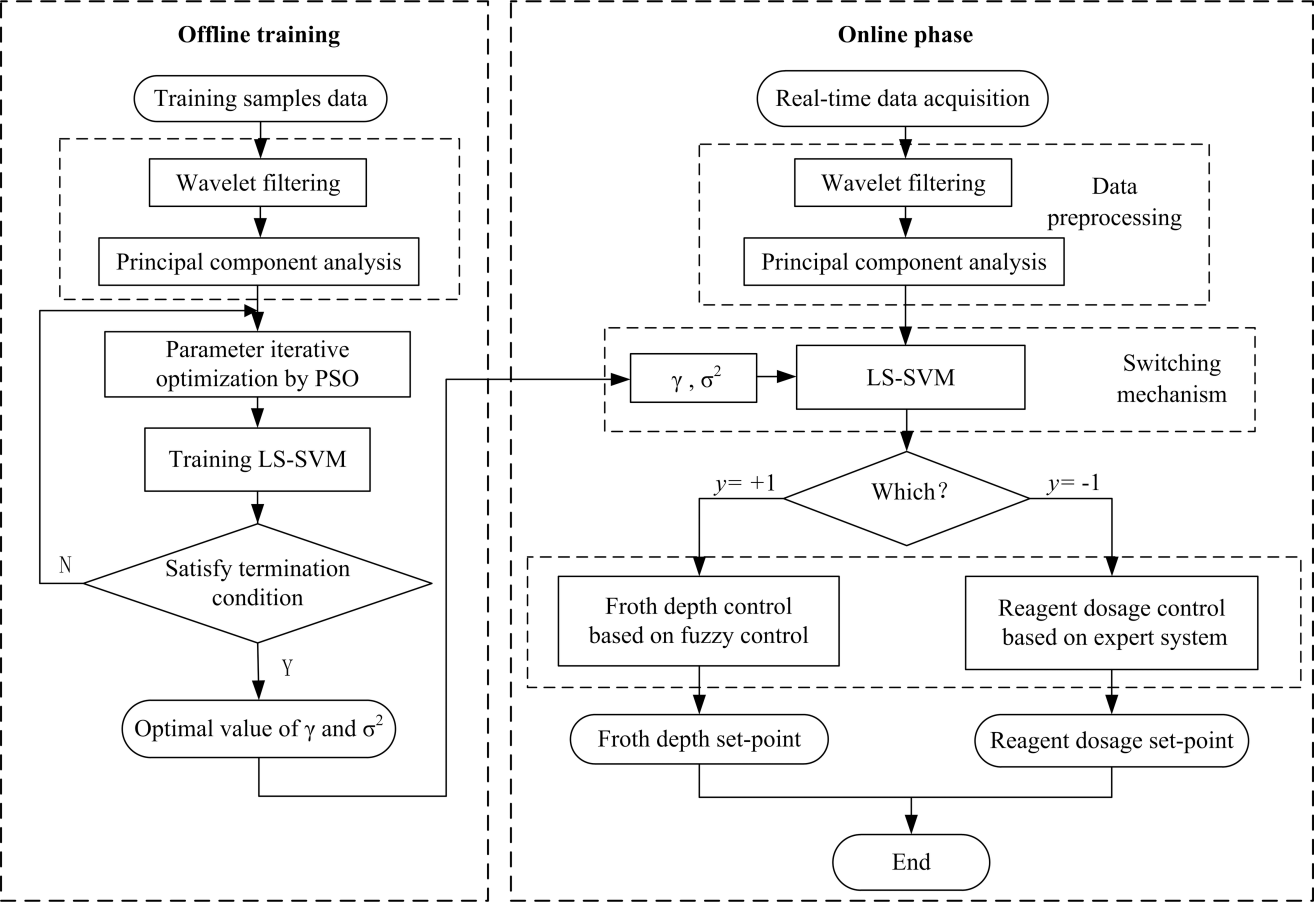
Computation flowchart of the hybrid model.

As described above, the computation flowchart of the hybrid model is shown in Fig 4.

## Implementation and evaluation

To validate its efficacy, the hybrid model is applied to an industrial flotation column in the Xingtai Coal Preparation Plant of Hebei Province, China. The flotation column is used in the separation of fine coal slime 0–0.25 mm in size. Kerosene is used as collector, and fusel is used as the frother. The experimental data are generated by the industrial process of the flotation column.

### Signal pre-processing

To eliminate the noise of the sensor in the industrial field and guarantee the quality of the sensor data, wavelet filtering is adopted [35]. In this work, the wavelet analysis method is used to filter the sensor signal. Because the signal obtained by the sensor is a discrete signal, the discrete wavelet transform [36] is adopted for signal processing.

The filter results should be evaluated with respect to the optimal filtering effect. In this work, the root-mean-square error (*RMSE*), signal-to-noise ratio (*SNR*) and smoothness are used.

The *RMSE* is defined as follows:

$$RMSE={\left\{{\left[f(n)-\hat{f}(n)\right]}^{2}/n\right\}}^{1/2}$$ (16)

where *f* (*n*) represents the original signal and *^f*(*n*) represents the signal after de-noising.

The *SNR* is defined as follows:

$$SNR=10{Log}_{10}(P_{s}/P_{z})$$ (17)

where *P*_*S*_ = [∑*f* ^2^(*n*)] **/***n* represents the original signal power and *P*_*S*_ = *RMSE*^2^ represents the noise power.

The smoothness index is defined as follows:

$$r=\left\{\sum_{n+1}{\left[\hat{f}(n+1)-\hat{f}(n)\right]}^{2}/\sum_{n+1}{\left[f(n+1)-f(n)\right]}^{2}\right\}$$ (18)

where *f* (*n*) represents the original signal and *^f*(*n*) represents the signal after de-noising.

The flotation feed flow rate *Q*, which is one of the input variables of the LS-SVM identification model, is used as an example of the wavelet filtering process. The Daubechies wavelet is adopted as the wavelet function, and the decomposition scale is 3. In MATLAB, the "wavedec" function is used in wavelet decomposition, the "Thselect" function is used in threshold selection, and the "wdencmp" function is used in wavelet reconstruction. Threshold selection plays an important role in the wavelet filtering process [37]. In this paper, the "rigrsure" rule based on the unbiased likelihood estimation, the "sqtwolog" rule based on the fixed threshold, the "heursure" method based on the heuristic threshold selection, and the "minimax" method based on the minimax principle are adopted as options for comparison in the threshold selection method to filter the original data. The original data are obtained using an electromagnetic flowmeter through a continuous sampling process with a sampling time interval of 1 s, yielding a total of 250 samples.

**Fig 5:**
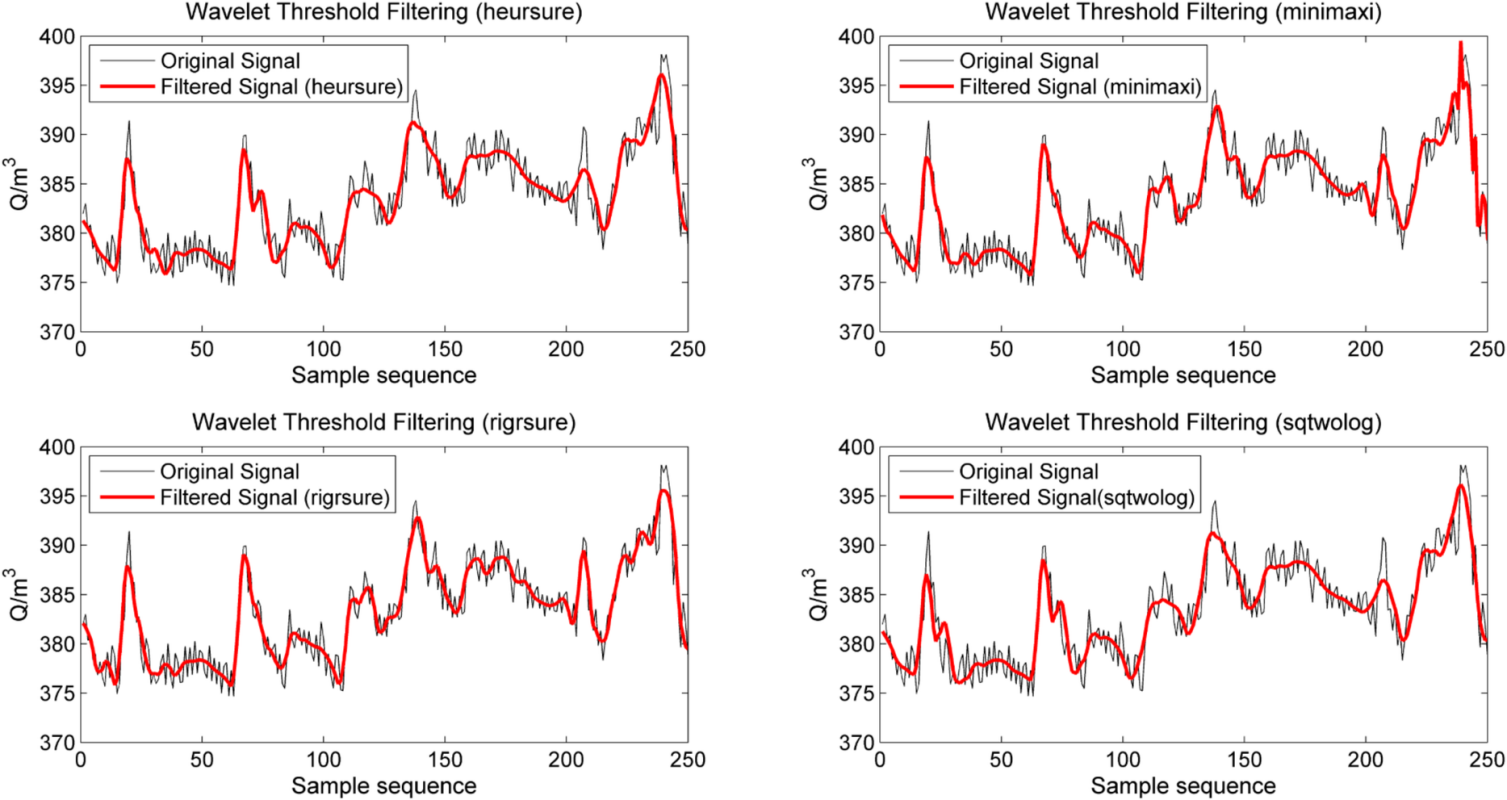
Comparison of the filtering effect.

The filtering results of the four threshold selection methods are shown in Fig 5. The results indicate that the stability and smoothness of the filtered signal are considerably better and that certain noise points have been eliminated. The evaluation of the four filter results is shown in Table 3.

**Table 3:** Evaluation index values of different threshold selection methods.

Threshold selection method	Evaluation index		
	*SNR*	*RMSE*	*r*
*heursure*	46.0665	1.9068	0.1117
*minimax*	47.3643	1.6422	0.2397
*rigrsure*	47.5049	1.6158	0.1506
*sqtwolog*	45.8228	1.9611	0.1128

Table 3 illustrates that the minimum *RMSE* and maximum *SNR* are obtained when the threshold selection is based on the "*rigrsure*" rule. Relative to the other three types of threshold selection methods, the noise reduction effect of the "*rigrsure*" rule is the best, and the deviation between the filtered data and original data is minimal. For the smoothness index *r*, the smoothness of the data filtered using the "*rigrsure*" rule is worse than when the "*heursure*" and "sqtwolog" rules are used but better than when the "*minimax*" rule is used. This result suggests that the wavelet threshold filtering method based on the "*rigrsure*" rule can capture the general trend of the original data while retaining more signal details. Considering the above analysis, the "*rigrsure*" rule, which is based on the unbiased likelihood estimation, is selected as the threshold determination method in this research.

Flotation is a complex process with numerous influence variables that are correlated to a certain degree. It is challenging to estimate which features are more sensitive to the model. To remove redundant information and reduce the computational complexity of the LS-SVM, PCA is used to extract features, fuse the correlation between variables and reduce the dimensions of the input data. For a coal preparation plant that washes a single type of coal, it is assumed that the variety of coal is stable and that the feed properties are therefore constant. Thus, the model input variables are composed predominantly of measurable parameters. Based on research on the mechanism of the coal slime flotation process and actual production experience, the primary measurable variables in the separation process of flotation column are shown in Table 4.

**Table 4:** Input variables of PCA.

Variable	Name
*C*	Feed concentration
*Q*	Feed flow rate
*P*	Circulating pulp pressure
*H*	Froth depth
*Qc*	Collector dosage
*Qf*	Frother dosage

**Fig 6:**
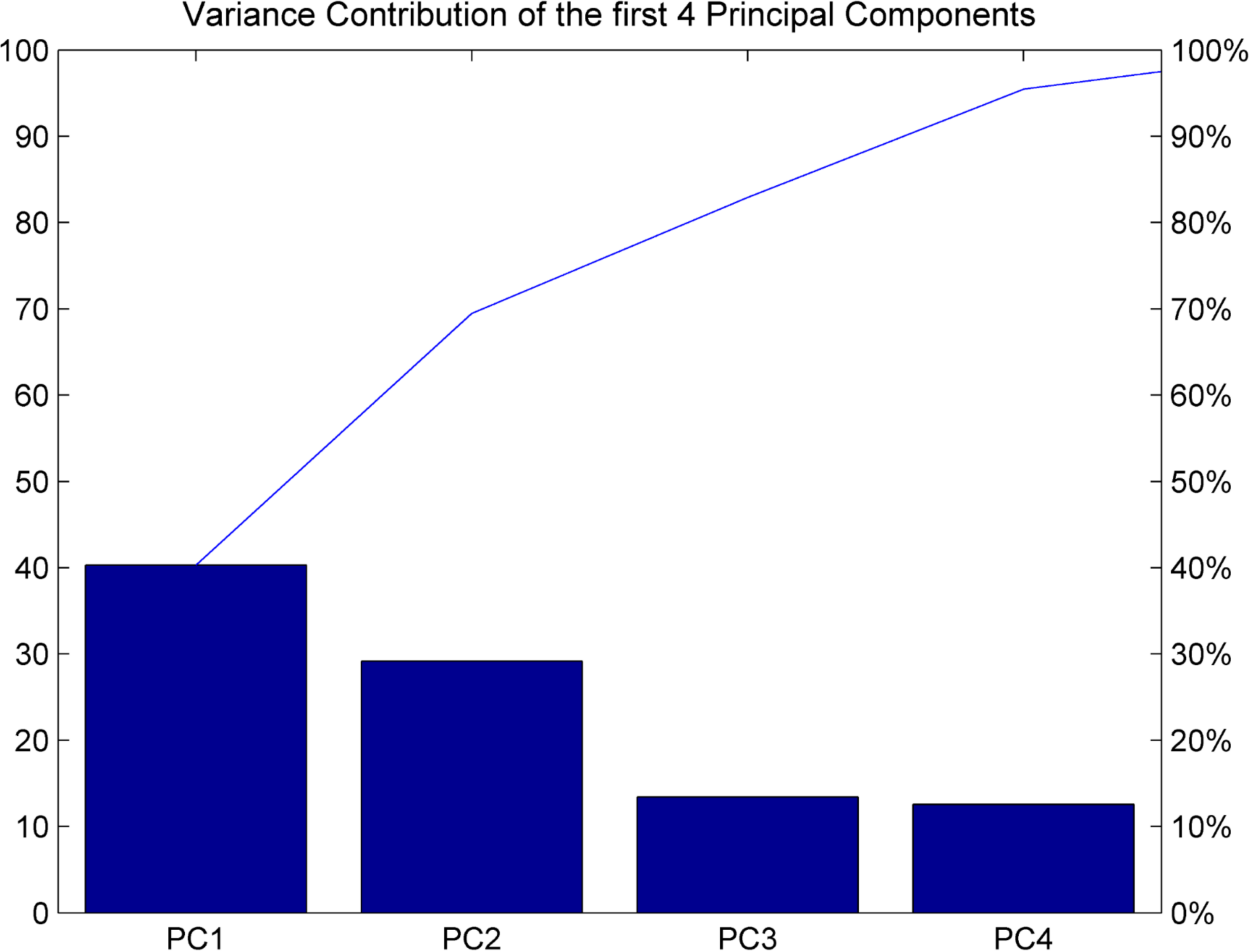
Schematic diagram of the principal component contributions.

In this research, 100 typical groups of data from the Xingtai Coal Preparation Plant from September to October 2014 that are filtered by the wavelet are selected. The data are analyzed by PCA. The variance contribution of each principal component is shown in Table 5. From Table 5, the cumulative contribution rate of the first four principal components is 95.47%, which is greater than 85% [38]. Therefore, the first four principal components shown in Fig 6 are selected as the input variables of the LS-SVM model, and the input/output relation can be expressed as in Eq (19):

y=sgnf(PC1,PC2,PC3,PC4) (19)

**Table 5:** Variance contribution of each principal component.

Principle component	Eigenvalue	Variance contribution/%	Accumulated contribution /%
1	0.1515	40.31	40.31
2	0.1097	29.18	69.49
3	0.0505	13.43	82.92
4	0.0472	12.55	95.47
5	0.0153	4.08	99.54
6	0.0017	0.46	100.00

### Prediction results and discussion

This paper establishes the identification and switching model based on the LS-SVM. The output *y*∈{−1,+1} is the identification result, where “*y = +*1” represents the froth depth control model and “*y =* -1" represents the reagent addition control model. The samples are selected from the historical operation data. When the froth depth begins to adjust, the current data set is labeled “+1”, and when the reagent dosage begins to adjust, the current data set is labeled “-1”. Ultimately, a total of 100 sets of samples are selected. Then, 80 of the sample sets are randomly selected as the training set, and the other 20 groups are used as the test set. The training set and test set are analyzed by PCA, and *PC1*, *PC2*, *PC3*, and *PC4* are selected as the input variables. The data sets are normalized to the range of 0–1 to eliminate the influence of different dimensions on the modeling process and increase the convergence rate of the model.

**Fig 7:**
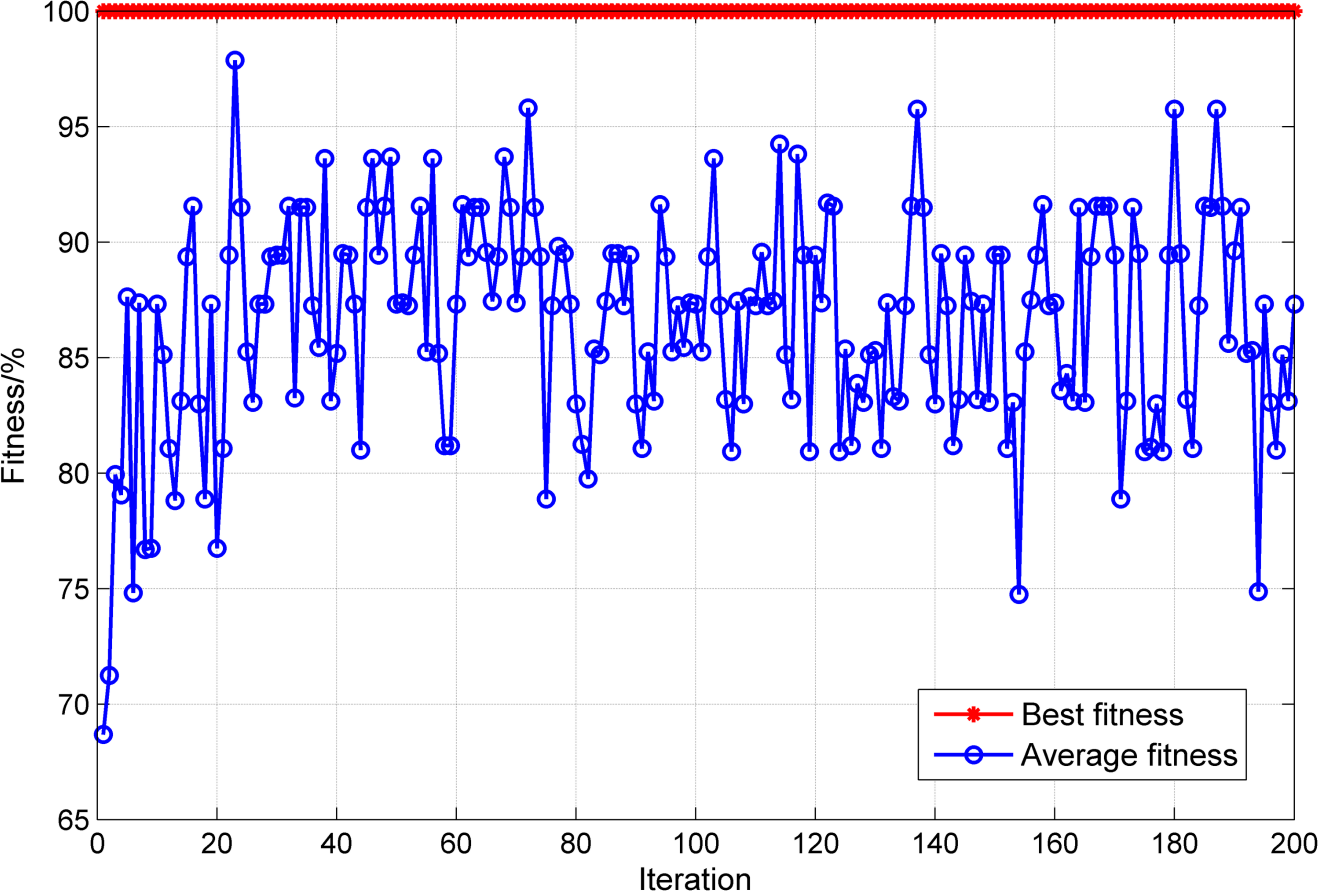
PSO optimization result.

**Fig 8:**
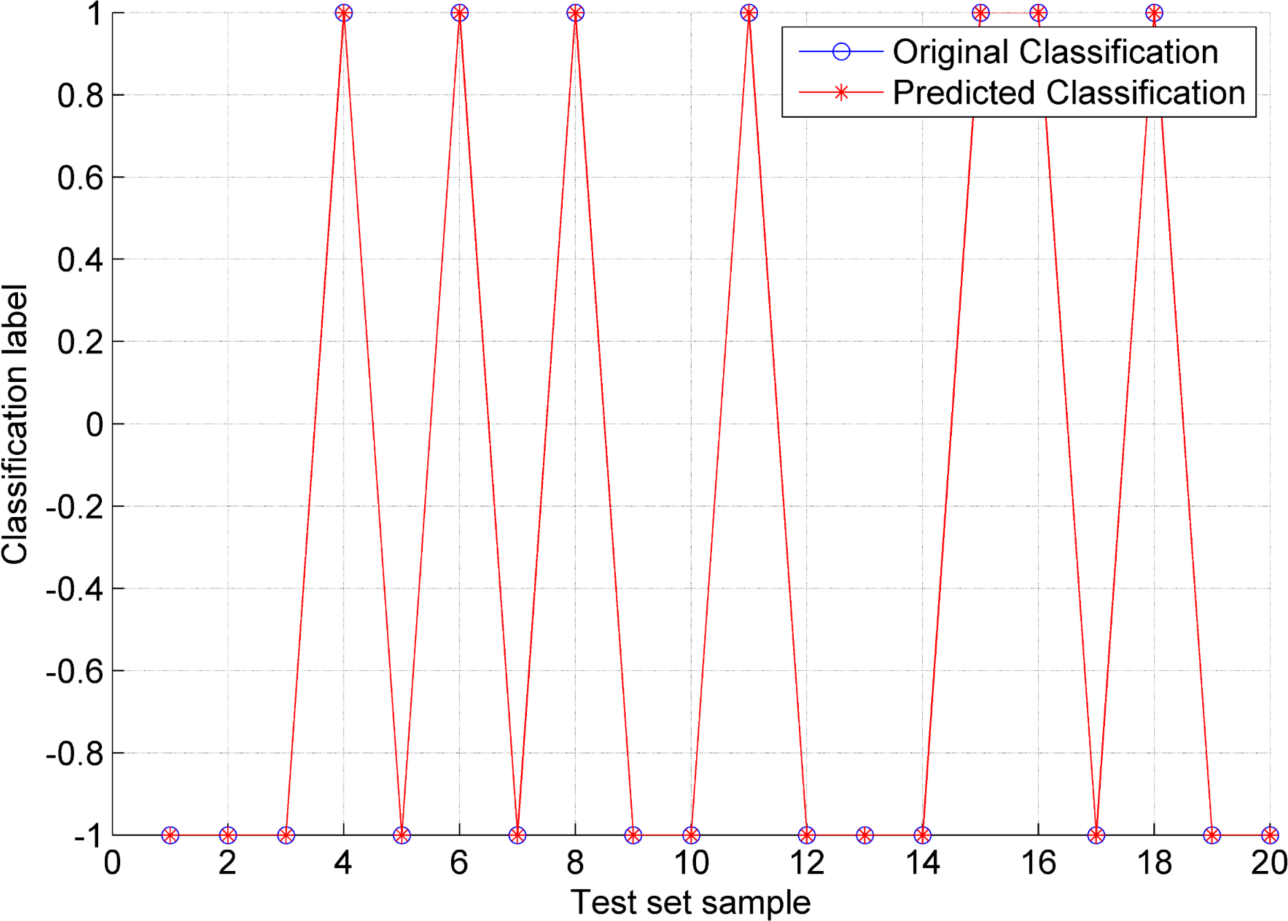
PSO-LSSVM classification result.

The LS-SVM model parameters are determined by the PSO algorithm, the population size is set to 20, the maximum number of iterations is set to 200, *c*_1_ is set to 1.7, *c*_2_ is set to 1.5, the initial value of the inertia factor ω is set to 1, ω_*max*_ is set to 1.2, ω_*min*_ is set to 0.8, γ_*min*_ is set to 0.01, γ_*max*_ is set to 1,000, σ^2^_*min*_ is set to 0.1, σ^2^_*max*_ is set to 100, and the cross-validation number is set to 3. The PSO results are shown in Fig 7. The optimized parameter γ is 0.01, and σ^2^ is 8.6397. The two optimized parameters are used to build the LS-SVM model. After the model is trained, the test samples are applied in testing, and the results are shown in Fig 8. The 20 groups of test samples are all correctly classified, and the accuracy of classification is 100%. The result shows that the classification model based on the LS-SVM performs well and can be used to identify the switching time of the control models during the flotation process.

To validate the proposed classification method in this paper, its performance was compared with that of other methods. The results are shown in Table 6.

**Table 6:** Comparison of classification accuracy for different models.

Method	Classification accuracy/%
Internal parameters of the LS-SVM model fixed by arbitrary selection	75 (15/20)
Internal parameters of the LS-SVM model fixed by PSO, without wavelet de-noising and PCA	95 (18/20)
Method proposed in this study	100 (20/20)

The classification accuracies of the different modeling methods differ considerably. If the parameters are selected arbitrarily, then the classification accuracy is notably poor. As shown in the table, the classification accuracy is only 75%, and the result has no predictive value. PSO is proven as a reasonable method for optimizing the internal parameters of the LS-SVM model. The noise in the original signal is significantly reduced by the wavelet transform, thus improving the robustness of the system. Simultaneously, PCA effectively extracts the main features and reduces the input dimensions, thereby ensuring identification accuracy. The proposed method in this research is best in terms of accuracy. By comparison, the proposed method performs effectively in identifying the switching points between different control models in the flotation process.

### Experiment and evaluation

**Fig 9:**
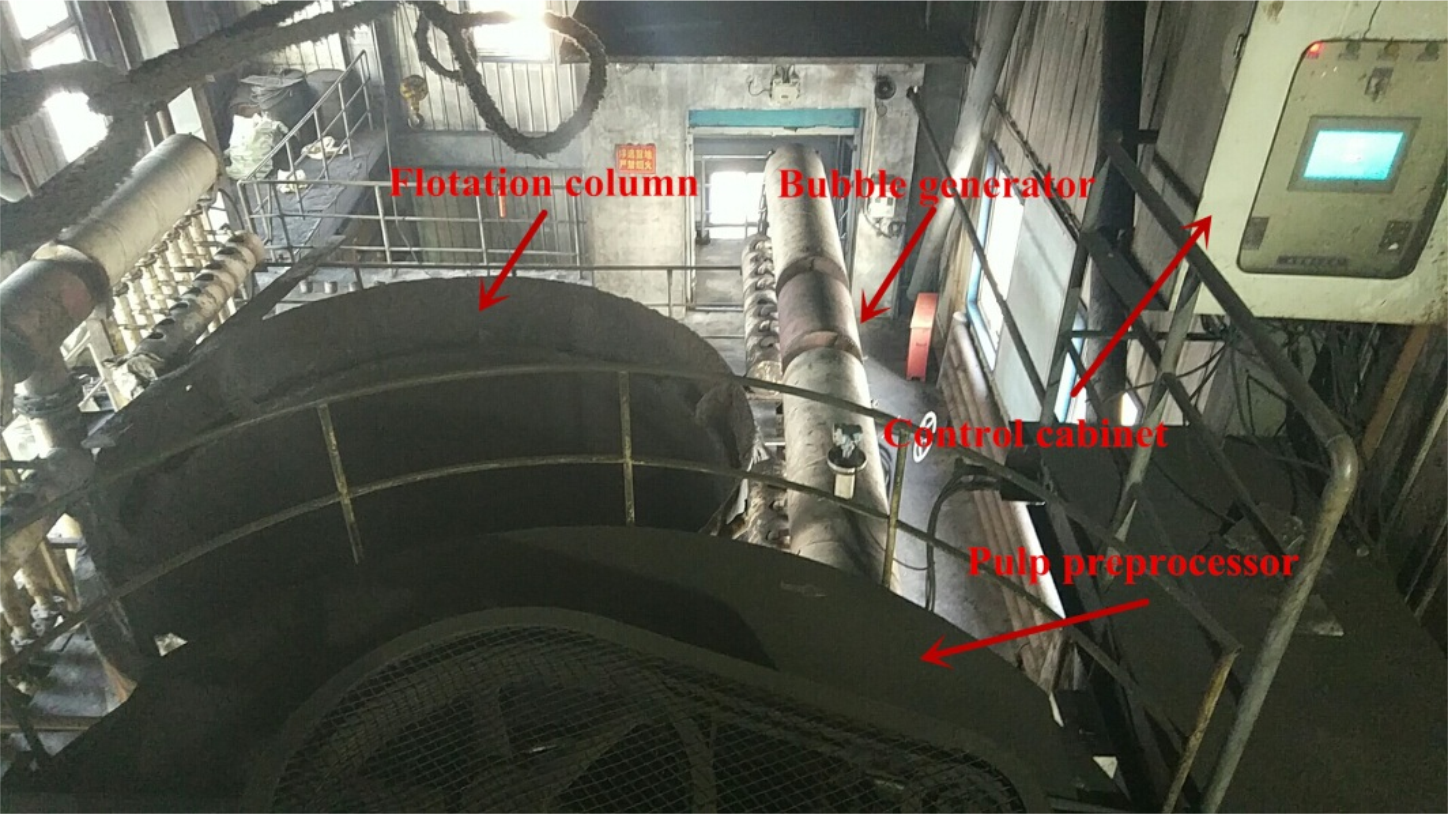
Diagram of the experimental FCMC-4500 flotation column.

A performance comparison was conducted between the #1 flotation column of the Xingtai Coal Preparation Plant under the control of the proposed hybrid model and the #2 flotation column under manual control for a period of 60 days. The two flotation columns were located in parallel, and their feeds originated from the same feed pipe such that the feed properties were considered completely consistent. Fig 9 shows the actual diagram of the FCMC-4500 flotation column used in the Xingtai Plant.

**Fig 10:**
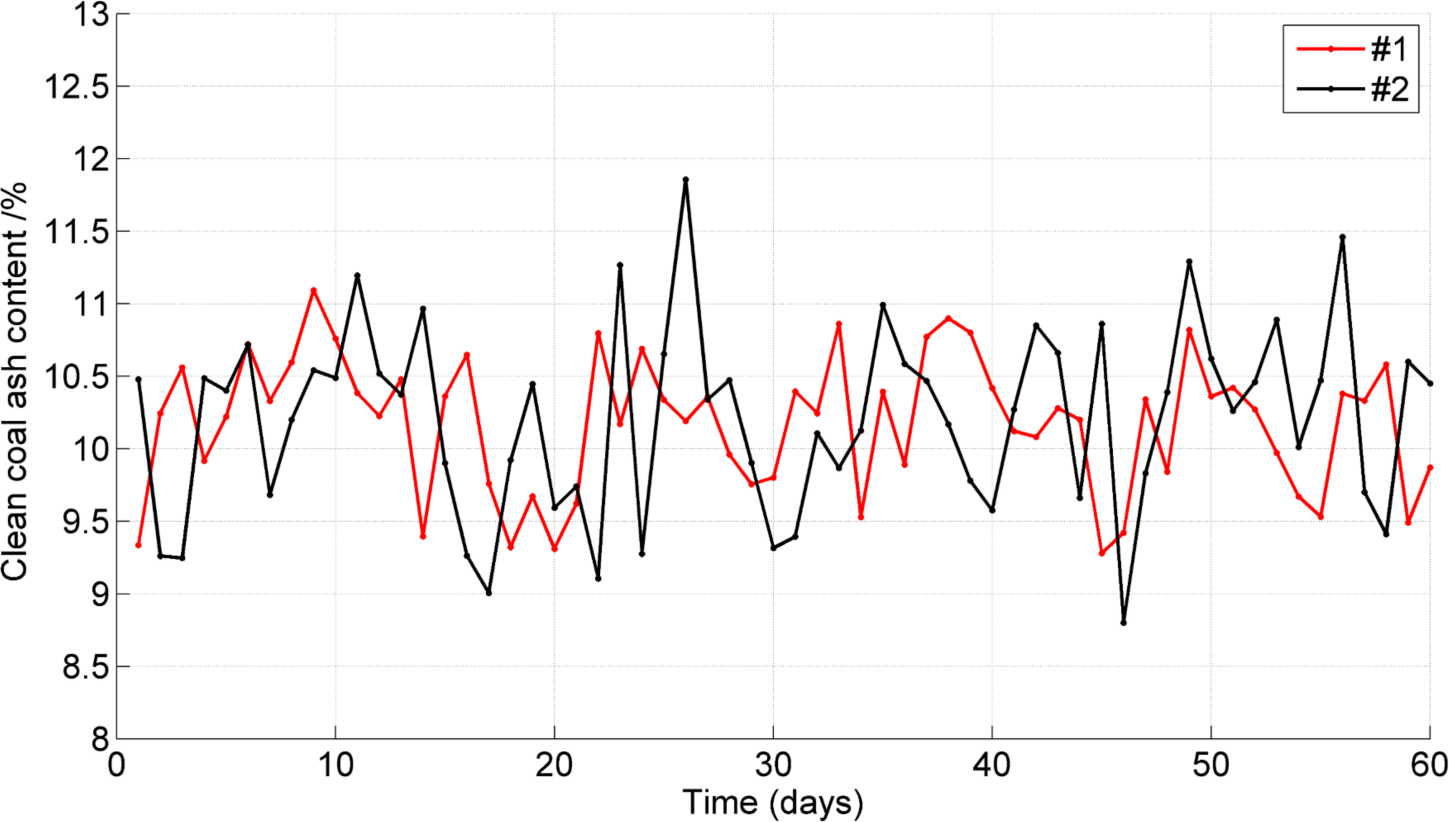
Comparison of clean coal ash content between the #1 and #2 flotation column.

**Fig 11:**
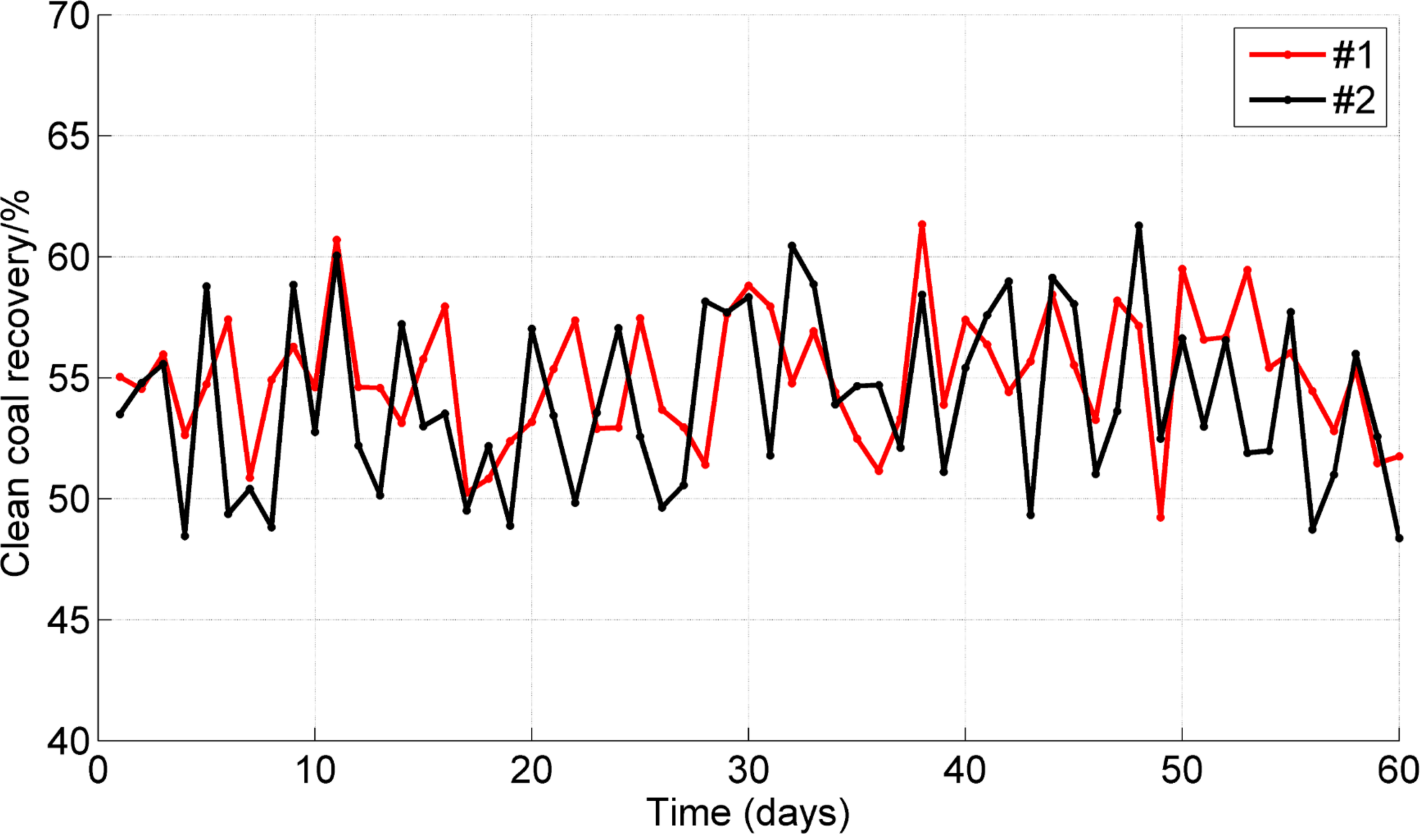
Comparison of clean coal recovery between the #1 and #2 flotation column.

**Fig 12:**
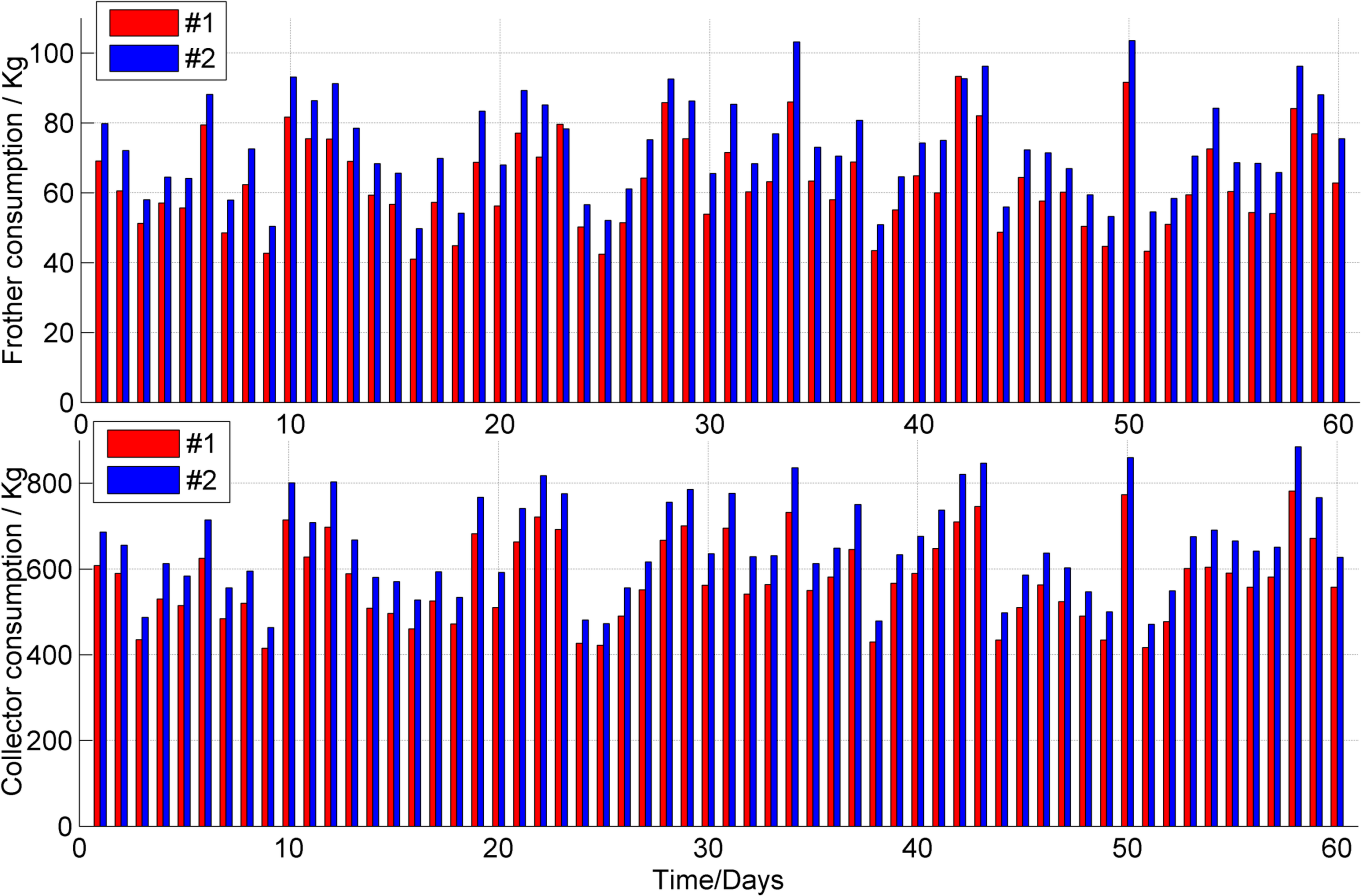
Comparison of reagents consumptions between the #1 and #2 flotation column.

During the 60 days, samples of clean coal from the flotation were collected every hour, providing a total of ten samples every day. The daily average ash content and recovery of clean coal are shown in Figs 10 and 11. The daily consumptions of the frother and collector are shown in Fig 12. The results show that the average clean coal ash content decreased from 10.21% to 10.17% and that its stability was improved, with a reduction in the standard deviation of the ash from 0.65% to 0.47%. The average clean coal recovery rate increased from 54.06% to 55.04%, and the standard deviation of the recovery was reduced from 3.59% to 2.64%. Furthermore, the average daily consumptions of the frother and collector were reduced by 14% and 12%, respectively.

## Conclusions

This paper describes a hybrid model for the coal flotation process. Wavelet analysis and PCA are used in signal pre-processing. The reagent dosage and froth depth are selected as the two manipulated variables, and the optimizing control model of froth depth based on fuzzy control and the optimizing control model for reagent addition based on an expert system are established. The hybrid model can execute a reasonable and timely switch of the controllers according to different condition parameters in the coal flotation process. The LS-SVM is used to identify the switching points between different control models. The internal parameters are optimized through PSO. The hybrid model was evaluated at the Xingtai Coal Preparation Plant. During the evaluation, the average clean coal ash content decreased from 10.21% to 10.17%, and its stability was improved, with a reduction in the standard deviation of the ash from 0.65% to 0.47%. The average clean coal recovery increased from 54.06% to 55.04%. The average daily consumptions of the frother and collector were reduced by 14% and 12%, respectively. This research offers an example of a hybrid intelligent control method for coal flotation columns.

## Supporting information

S1 File Summary of evaluation results data. (PDF)
