# Supplementary material for: Switching and optimizing control for coal flotation process based on a hybrid model
Source: PLoS One. 2017 Oct 17;12(10):e0186553. doi: 10.1371/journal.pone.0186553 (PMC5645138; doi:10.1371/journal.pone.0186553)
Supplement: S1 File — (PDF) [file pone.0186553.s001.pdf]

Sequence	Recovery/%		Clean coal ash content/%	
	#1	#2	#1	#2
1	55.04	53.50	9.34	9.34
2	54.54	54.79	10.24	10.24
3	55.96	55.57	10.56	10.56
4	52.64	48.47	9.92	9.92
5	54.74	58.78	10.22	10.22
6	57.41	49.38	10.72	10.72
7	50.87	50.41	10.33	10.33
8	54.92	48.83	10.60	10.60
9	56.28	58.84	11.09	11.09
10	54.61	52.77	10.76	10.76
11	60.70	60.05	10.38	10.38
12	54.62	52.20	10.23	10.23
13	54.58	50.14	10.48	10.48
14	53.14	57.22	9.40	9.40
15	55.78	53.00	10.36	10.36
16	57.95	53.52	10.65	10.65
17	50.26	49.52	9.76	9.76
18	50.83	52.18	9.32	9.32
19	52.38	48.89	9.67	9.67
20	53.18	57.03	9.31	9.31
21	55.36	53.44	9.62	9.62
22	57.37	49.84	10.80	10.80
23	52.90	53.56	10.17	10.17
24	52.95	57.06	10.69	10.69
25	57.46	52.58	10.34	10.34
26	53.69	49.65	10.19	10.19
27	52.96	50.57	10.36	10.36
28	51.41	58.16	9.96	9.96
29	57.63	57.71	9.75	9.75
30	58.81	58.34	9.80	9.80
31	57.95	51.80	10.39	10.39
32	54.77	60.46	10.24	10.24
33	56.93	58.87	10.86	10.86
34	54.42	53.90	9.53	9.53
35	52.48	54.66	10.39	10.39
36	51.15	54.70	9.89	9.89
37	53.33	52.12	10.77	10.77
38	61.34	58.44	10.90	10.90
39	53.89	51.11	10.80	10.80
40	57.40	55.41	10.42	10.42

41	56.37	57.59	10.12	10.12
42	54.42	58.99	10.08	10.08
43	55.69	49.34	10.28	10.28
44	58.45	59.14	10.20	10.20
45	55.53	58.05	9.28	9.28
46	53.26	51.03	9.42	9.42
47	58.19	53.63	10.34	10.34
48	57.15	61.29	9.84	9.84
49	49.23	52.48	10.82	10.82
50	59.50	56.63	10.36	10.36
51	56.58	52.98	10.42	10.42
52	56.68	56.55	10.27	10.27
53	59.46	51.90	9.97	9.97
54	55.41	51.98	9.67	9.67
55	56.03	57.72	9.53	9.53
56	54.46	48.73	10.38	10.38
57	52.80	51.00	10.33	10.33
58	55.38	55.99	10.58	10.58
59	51.48	52.57	9.49	9.49
60	51.75	48.38	9.87	9.87

Sequence	Daily consumptions of reagents/Kg			
	Frother/Kg		Collector/Kg	
	#1	#2	#1	#2
1	69.05	79.75	608.39	685.90
2	60.46	72.07	589.56	655.80
3	51.22	57.95	434.71	486.80
4	57.09	64.52	530.16	612.90
5	55.69	64.15	514.82	583.70
6	79.38	88.20	625.10	714.40
7	48.51	57.89	484.01	555.70
8	62.26	72.55	519.94	594.90
9	42.68	50.40	415.48	463.70
10	81.69	93.14	714.49	801.00
11	75.48	86.35	628.08	708.10
12	75.38	91.25	697.81	803.00
13	69.03	78.52	588.65	667.40
14	59.29	68.31	508.26	580.20
15	56.69	65.61	496.03	570.80
16	41.04	49.73	460.33	527.30
17	57.27	69.84	525.34	593.60
18	44.86	54.20	472.14	534.10
19	68.72	83.41	682.22	767.40
20	56.19	68.03	510.22	591.90
21	77.12	89.26	663.11	740.90
22	70.21	85.21	721.48	818.00
23	79.56	78.32	692.34	775.30
24	50.22	56.62	426.43	481.30
25	42.39	52.10	422.30	472.90
26	51.38	61.10	490.39	556.00
27	64.20	75.18	551.68	616.40
28	85.77	92.58	667.02	755.40
29	75.46	86.34	700.84	785.70
30	53.87	65.53	562.51	635.60
31	71.48	85.29	695.48	776.20
32	60.34	68.33	541.31	628.70
33	63.23	76.93	563.94	630.80
34	85.99	103.23	731.68	836.20
35	63.40	72.96	549.77	612.90
36	58.03	70.52	581.32	648.80
37	68.82	80.68	645.26	750.30
38	43.35	50.94	429.96	478.80
39	55.14	64.64	566.89	633.40

40	64.92	74.28	589.47	676.00
41	59.98	74.97	648.00	737.20
42	93.26	92.74	709.99	820.80
43	82.14	96.19	745.77	846.50
44	48.72	55.94	434.08	497.80
45	64.37	72.33	510.32	585.90
46	57.56	71.40	563.02	636.90
47	60.25	66.94	524.09	602.40
48	50.38	59.42	489.75	546.60
49	44.69	53.20	434.09	500.10
50	91.60	103.62	773.23	860.10
51	43.30	54.48	416.63	471.30
52	50.96	58.38	477.46	548.80
53	59.43	70.48	601.20	675.50
54	72.59	84.20	604.10	690.40
55	60.37	68.61	590.30	665.50
56	54.32	68.40	557.81	641.90
57	54.05	65.76	581.34	651.00
58	84.10	96.23	781.81	885.40
59	76.93	88.13	671.63	766.70
60	62.78	75.53	557.31	626.90
